# Association Lp-PLA2 Gene Polymorphisms with Coronary Heart Disease

**DOI:** 10.1155/2022/9775699

**Published:** 2022-07-02

**Authors:** Sha Ma, Liangcai Ding, Mengdi Cai, Lu Chen, Bo Yan, Jian Yang

**Affiliations:** ^1^Department of Cardiology, Jining First People's Hospital, Jining Medical University, Jining, Shandong 272000, China; ^2^Center for Molecular Medicine, Yanzhou District People's Hospital, Jining, Shandong 272100, China; ^3^The Center for Molecular Genetics of Cardiovascular Disease, Affiliated Hospital of Jining Medical University, Jining Medical University, Jining, Shandong 272029, China; ^4^Division of Cardiology, Yanzhou District People's Hospital, Jining, Shandong 272100, China

## Abstract

**Objectives:**

The study evaluated the association between lipoprotein-associated phospholipase A2 (Lp-PLA2) gene polymorphisms and coronary heart disease (CHD), in order to explore the molecular genetics of CHD.

**Methods:**

Groups of CHD patients (*n* = 283) and healthy controls (*n* = 261) were involved in this study. R92H, V279F, and A379V polymorphisms of LP-PLA2 gene were confirmed using polymerase chain reaction (PCR) and direct DNA sequencing. These polymorphisms and their interaction were also analyzed as potential risk factors of CHD.

**Results:**

In this study population, the genotypes of R92H (GG, GA, and AA), V279F (CC, AC, and AA) and A379V (GG, GA, and AA) were studied. There was a significantly difference in frequencies of R92H between CHD patients and controls (*P* < 0.05). In contrast, no significant difference in frequencies of V279F and A379V existed between CHD patients and controls. Furthermore, R92H and A379V were in strong linkage disequilibrium.

**Conclusions:**

These results suggested that R92H polymorphism might contribute to increased risk of CHD.

## 1. Introduction

Coronary heart disease (CHD, also known as coronary atherosclerotic heart disease) is the leading cause of death in most industrialized countries. Atherosclerosis has a strong inflammatory response [1]. Accumulating studies have supported the role of inflammatory response in the development of CHD [2]. Inflammation occurs at all stages of coronary atherosclerosis and is highly correlated with the accumulation of lipids. In recent years, the role of lipoprotein-associated phospholipaseA2 (Lp-PLA2) in CHD has been focused. Lp-PLA2 is an enzyme participating in lipoprotein metabolism and inflammatory pathways. Lp-PLA2 generates proinflammatory and proatherogenic compounds, emerging as a potential therapeutic target for CHD.

**Figure 1:**
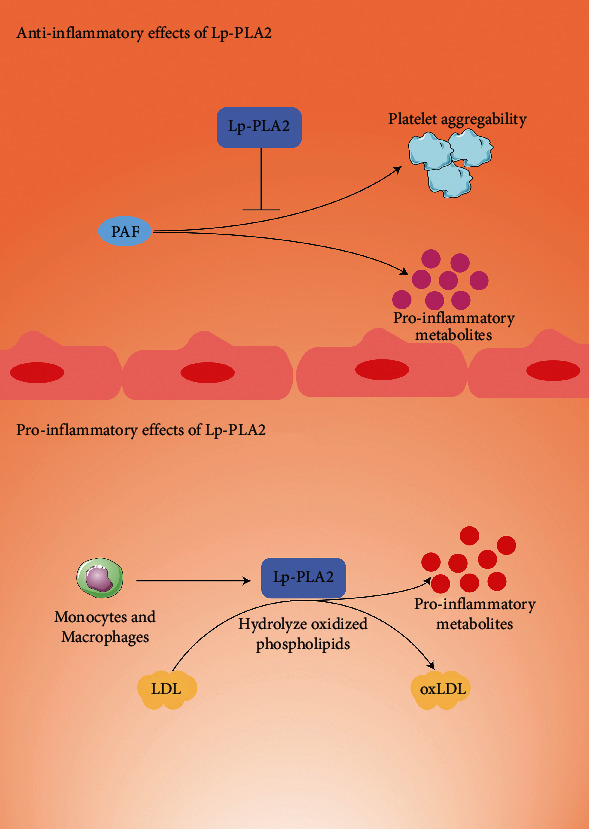
The anti-inflammatory effect and proinflammatory effect of Lp-PLA2.

Lp-PLA2 gene is located at 6P21.2-P12, contains 12 exons. Lp-PLA2, also known as platelet-activating factor acetylhydrolase (PAF-AH), is a 45.4 kD calcium-independent member of the phospholipase A2 family. As shown in Figure 1, inflammation plays an important role in the pathogenesis of vascular diseases [3], and Lp-PLA2 is produced by the inflammatory cell in atherosclerotic plaque [4]. Epidemiological studies have shown dual-roles of Lp-PLA2. On one hand, Lp-PLA2 was recognized for its action in hydrolyzing platelet-activating factor [5]. On the other hand, Lp-PLA2 is mainly produced by monocytes and macrophages and can hydrolyze oxidized phospholipids, produce oxidative modification of low-density lipoprotein, and release proatherogenic and proinflammatory metabolites [6]. Small portion of circulating Lp-PLA2 enzyme (20%) is associated with high-density lipoprotein (HDL), while 80% of Lp-PLA2 enzyme is associated with low-density lipoprotein (LDL) [7]. Recent studies have reported that the level and activity of Lp-PLA2 are related to the risk of cardiovascular events [8–10], and high Lp-PLA2 activity implies a worse CV prognosis [11]. Heritability studies revealed that approximately 62% of the variation in Lp-PLA2 activity was related with genetic factors [12].

Altered Lp-PLA2 activity relevant to nonfunctional V279F allele has been reported in a Japanese population. Plasma Lp-PLA2 activity is absent in homozygous V279F carriers, and plasma LP-PLA2 activity is reduced by 50% in heterozygous V279F carriers [13]. In western countries, frequency of V279F genotype is presented with a gradient descent. Frequency of V279F genotype in China and South Korea is in the middle. V279F is relatively rare in the Middle East and almost absent in the European population [14]. A relationship between V279F and Asians with CHD has been reported. V279F allele increases the risk of myocardial infarction and stroke in a Japanese population [15, 16]. Subsequent studies have obtained contrary association results in the Japanese or Chinese population [17, 18]. V279F allele carriers have reduced risk of CHD in the Korean male population [19]. A92H and V379A alleles are also founded in European populations [20–22]. A379V allele lowers the risk of myocardial infarction. Similarly, the results from a number of studies for R92H and A379V are not consistent. Therefore, the difference of study remains to be further explored.

Although numerous studies have evaluated the association between Lp-PLA2 gene polymorphisms and coronary heart disease, the conclusions are still inconsistency. Here we examined the correlation between R92H, V279F, and A379V polymorphism of Lp-PLA2 and coronary heart disease (CHD). Further, its clinical value was assessed as biomarkers. Three SNPs of LP-PLA2 gene, R92H, V279F, and A379V, have been associated with CHD in different populations. In this study, we aimed to associate these SNPs in Lp-PLA2 gene with CHD in a Chinese population.

## 2. Materials and Methods

### 2.1. Study Design and Participants

We recruited 283 unrelated Han Chinese patients with CHD between December 2017 and October 2018 at Yanzhou Hospital, Affiliated Hospital of Jining Medical University, Shandong, China. All CHD patients were confirmed by coronary angiography. The healthy control group consisted of 261 unrelated subjects for routine health examinations in the same hospital. All controls had no history of cerebrovascular disease, severe hepatic, or renal disease. Informed consents were signed by all participants. This study was approved by the Ethics Committee of the hospital.

### 2.2. Clinical Samples

Peripheral venous blood was collected. Automatic biochemical analyzer was used to measure the full set of blood lipids, including total cholesterol (TC), triglycerides (TG), high-density lipoprotein (HDL), and low-density lipoprotein (LDL). Clinical history for smoking, drinking, diabetes, and high blood pressure was documented.

#### 2.2.1. Analysis of Lp-PLA2 Gene Polymorphisms

**Figure 2:**
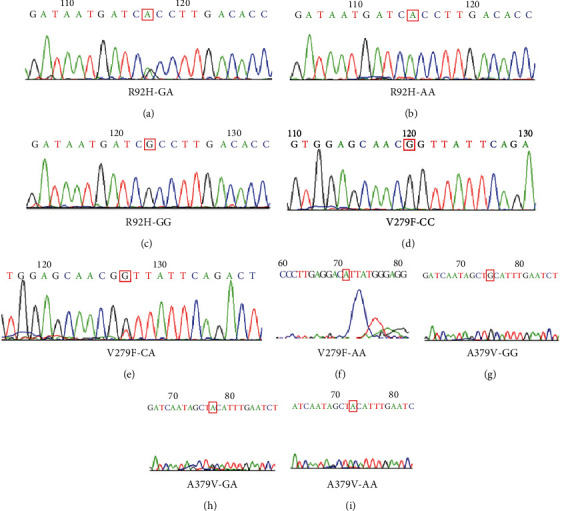
Sequencing chromatograms for R92H, V279F, and A379V in the Lp-PLA2 gene. (a) Genotype GA of R92H. (b) Genotype AA of R92H. (c) Genotype GG of R92H. (d) Genotype CC of V279F. (e) Genotype CA of V279F. (f) Genotype AA of V279F. (g) Genotype GG of A379V. (h) Genotype GA of A379V. (i) Genotype AA of A379V.

Genomic DNAs were extracted from peripheral leukocytes and examined with electrophoresis. These genotypes (R92H, V279F, and A379V) were confirmed by PCR and direct DNA sequencing. PCR primers and PCR products are summarized in Table 1. PCR conditions were the following: denaturing at 95°C for 5 min and then repeating the following 25 cycles, denaturing at 95°C for 30 seconds, annealing at 58°C for 45 seconds, and extending at 72°C for 1 minute with a final extension of 7 minutes. PCR products were bidirectionally sequenced (Sangon Biotech Co., Shanghai, China). These genotypes were then confirmed as shown in Figure 2.

### 2.3. Statistical Analysis

Data of frequencies such as sex, smoking, drinking, high blood pressure, and diabetes were compared by *χ*^2^ test. Quantitative data such as age, total cholesterol, triglycerides, HDL cholesterol, LDL cholesterol, and lipoprotein A was expressed with mean ± SD and analyzed with Student's *t*-test. Genotype frequencies were compared between the CHD group and the control group. Hardy-Weinberg equilibrium was confirmed in this study population. Unconditional logistic regression for the relationship between Lp-PLA2 genotype and CHD was performed with degree of association, odds ratio, and 95% confidence interval. Coefficient of linkage disequilibrium and haplotype analyzes were performed using the SHEsis software. The SPSS22.0 software was used for statistical analysis.

## 3. Results

### 3.1. Clinical and Biochemical Characteristics

The clinical and biochemical characteristics of CHD patients and controls are summarized in Table 2. Majority of CHD patients were males. Ages of CAD cases were older than controls. Frequency of hypertension and prevalence of diabetes in CHD patients were significantly higher than those in controls, respectively (*P* < 0.001 and *P* < 0.001). HDL-C level was significantly lower in CHD patients than that in controls (*P* < 0.001). There was no significant difference in frequencies of drinking and smoking between the two groups.

### 3.2. Distribution of the Lp-PLA2 Gene Genetic Variants in CHD Patients and Controls

Distribution of the three variants of Lp-PLA2 gene in CHD patients and controls is summarized in Table 3. The genotype frequency distributions of the *Lp-PLA2* rs1805017 polymorphisms were 69.96% (GG), 24.73% (GA), and 5.30% (AA) in the CHD group and 75.48% (GG), 22.99% (GA), and 1.53% (AA) in the control group. The genotype frequency distributions of the *Lp-PLA2* rs76863441 polymorphisms were 88.69% (CC), 10.95% (CA), and 0.35% (AA) in the CHD group and 92.72% (CC), 5.75% (CA), and 1.53% (AA) in the control group. The genotype frequency distributions of the *Lp-PLA2* rs1051931 polymorphisms were 70.32% (GG), 27.21% (GA), and 2.47% (AA) in the CHD group and 67.05% (GG), 29.12% (GA), and 3.83% (AA) in the control group. For R92H, the frequencies of allele A were significantly higher in CHD patients than those in controls (*P* < 0.05). In contrast, there was no significant difference in frequencies of genotypes V279F and A379V between CHD patients and controls (*P* > 0.05).

### 3.3. Linkage Disequilibrium and Haplotypes of Lp-PLA2 Gene

**Figure 3:**
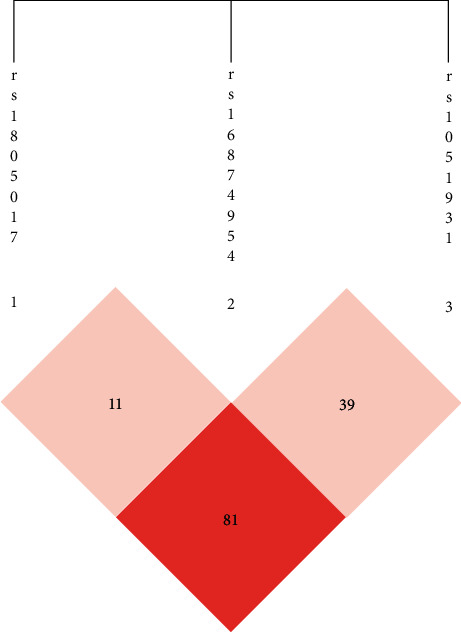
Linkage disequilibrium and haplotypes of Lp-PLA2 gene (1).

As shown in Figure 3, R92H and A379V were in strong linkage disequilibrium. Haplotype analysis with the SHEsis software revealed the genetic variants (R92H and A379V) formed four types of haplotypes (Table 4). There is a significant difference between the distribution of haplotypes (R92H and A379V) in CHD patients and controls (*P* < 0.05).

### 3.4. Association of Genetic Variants and Serum Lipid Levels

Association of serum lipid levels (TC, TG, HDL, and LDL) with these genetic variants was analyzed in CHD patients and controls. There was no significant difference between genotypes and levels of TG and LDL in CHD patients and controls. TC levels were significantly elevated in subjects with AC genotype of V279F in CAD patients than in those with CC genotype.

### 3.5. Logistic Regression Analysis

Logistic regression analysis showed that after adjustment for age, gender, smoking, history of diabetes, serum TC, TG, HDL-C, and LDL, V279F, A379V, and CHD had no correlation (Table 5). Sex, smoking, drinking, hypertension, and diabetes are independent risk factors for coronary heart disease. Collectively, R92H in Lp-PLA2 gene may contribute to CHD as a risk factor.

## 4. Discussion

Phospholipase A2 is a phospholipase that can be secreted into the circulation or becomes lipoprotein associated (Lp-PLA2), with the latter mostly bound to LDL. Lp-PLA2 hydrolyzes oxidized phospholipids to produce proinflammatory products that are implicated in endothelial dysfunction, plaque inflammation, and formation of a necrotic core in atherosclerotic plaque.

CHD has been regarded as one of the leading health threats in China. It is a multifactorial disease, genetic, and environmental factors, and their interactions also contribute to the development of CHD [23]. The activity, mass, and distribution of the Lp-PLA2 are associated with atherosclerosis and inflammatory diseases [24, 25]. An association between the variety of Lp-PLA2 mass and activity and CHD has been reported [26]. Lp-PLA2 plays an important role in the pathogenesis of CHD. Lp-PLA2, a novel inflammatory biomarker, is an independent risk predictor for cardiovascular disease [27]. The inflammatory response is not only the initial factor of CHD but is also involved in all stages of clinical atherosclerotic disease. Lp-PLA2 gene polymorphism is closely related to CHD, providing new clues for further understanding of the mechanism and prevention of development of CHD.

Three genotypes of R92H are GG, GA, and AA. Our study showed that AA genotype and A allele were greater in the CHD group than the control group, which were consistent with Tuten et al. [28]. Sutton et al. confirmed G allele increased in the CHD group [29]. The difference between the results may be due to ethnic heterogeneity.

The variant V279F of the Lp-PLA2 gene has been reported with various results in Indonesia, China, Korea, Japan, and Caucasian populations [30]. The V279F site has three genotypes, CC, AC, and AA. In this study, there is no statistical significance in genotypes of V279F between CHD and control groups (*P* > 0.05). Our results were not consistent with a comprehensive meta-analysis of LP-PLA2 gene V279F polymorphism [31].

In this study, the A379V site has GG, GA, and AA genotypes. Frequency of AA genotype was smaller in the CHD group than the control group. The genotype and allele proportion of A379V in the CHD group and the control group were similar (*P* > 0.05). The result of this study was inconsistent with Chinese Han population study by Li et al. [32]. Li et al.'s study indicated that AA genotype can increase the risk of myocardial infarction, whereas our results suggested that AA genotype is a protective factor.

R92H site and A379V site present linkage disequilibrium. Lp-PLA2 gene can form four types of haplotypes: A-A, A-G, G-A, and G-G. The distribution of haplotypes of A-G in CHD and control groups has significant difference. Hoffmann et al. analyzed five polymorphisms (-1357G>A, -403T>C, Arg92His, Ile198Thr, and Ala379Val) and haplotypes of Lp-PLA2 gene [33], long-term survival, and plasma Lp-PLA2 activity of Caucasian patients with CHD. They have found that 403C and 92H are associated with the reduction of plasma Lp-PLA2 activity, and A379V is associated with the elevation of plasma Lp-PLA2 activity. Ile198Thr is not correlated with plasma Lp-PLA2 activity. These five variants have no significant correlation with CAD. But the correlation between these polymorphisms and cardiovascular disease in other populations is still not clear. In order to further explore this issue, we also need to investigate other mutants of Lp-PLA2 gene. In addition, we also compared serum lipid levels of the different genotype between the CHD group and the control group, and the result showed that the serum HDL level was affected by the gene variant.

Lp-PLA2 gene is involved in many diseases, including acute pancreatitis and migraine without aura [34, 35], as well as atherosclerosis [36]. In this study, we reported that the R92H gene polymorphisms were associated with CHD. Though these data were not consistent with previous studies, our findings may provide an insight into our understanding the CHD pathogenesis. As inflammation is involved in the CHD development, it is also considered as a therapeutic target in atherosclerosis [37]. The limitation of this study was small sample size. Further understanding the mechanism of Lp-PLA2 gene in the CHD with large cohort of samples may provide new strategies for the prevention and treatment of CHD.

## Figures and Tables

**Table 1:** PCR primers for Lp-PLA2 gene.

PCR primers	DNA sequences	PCR products
R92H (rs1805017)	F: 5′-ACAGAGGTATTTGAGTCCCCAC-3′	231 bp
	R: 5′-AATGTTGCCCATAAGCCAGT-3′	
V279F (rs76863441)	F: 5′-TCTTATTTTCTTACCTGAATCTCTGA-3′	200 bp
	R: 5′-CATCCCCATGAAATGAACAAT-3′	
A379V (rs1051931)	F: 5′-TTTGTCCTGAGATTCATCTGGTT-3′	159 bp
	R: 5′-ACTGGCAAAATAATTGGACACA-3′	

**Table 2:** Clinical and biochemical characteristics of CHD patients and controls.

Characteristics	CHD (*n* = 283)	Controls (*n* = 261)	*P*值
Male, *n* (%)	192 (68%)	155 (59%)	0.0490
Age, years	65.16 ± 10.18	63.11 ± 10.54	0.0220
Smoking (*n*, %)	96 (34%)	71 (27%)	0.0950
Drinking (*n*, %)	58 (20%)	65 (25%)	0.2590
Hypertension (*n*, %)	161 (57%)	68 (26%)	<0.001
Diabetes (*n*, %)	73 (26%)	6 (2.0%)	<0.001
TC (mmol/L)	4.24 ± 1.06	4.41 ± 1.24	0.0736
TG (mmol/L)	1.41 ± 1.20	1.29 ± 0.75	0.1571
HDL-C (mmol/L)	1.17 ± 0.36	1.46 ± 0.30	<0.0001
LDL-C (mmol/L)	2.53 ± 0.87	2.43 ± 0.75	0.1547

**Table 3:** Genotype and allele frequencies in the CHD group with the control group.

Variants	Genotypes	CHD (*n* = 283, %)	Controls (*n* = 261, %)	OR	95% low	95% high	*P*值
R92H	GG GA AA	198 (69.96%) 70 (24.73%) 15 (5.30%)	197 (75.48%) 60 (22.99%) 4 (1.53%)	1.321	0.904	1.932	0.1496
	G A	466 (82.33%) 100 (17.67%)	454 (86.97%) 68 (13.03%)	1.433	1.026	2.001	0.0343
V279F	CC CA AA	251 (88.69%) 31 (10.95%) 1 (0.35%)	242 (92.72%) 15 (5.75%) 4 (1.53%)	1.624	0.896	2.943	0.1073
	C A	533 (94.17%) 33 (5.83%)	499 (95.59%) 23 (4.41%)	1.343	0.778	2.319	0.2881
A379V	GG GA AA	199 (70.32%) 77 (27.21%) 7 (2.47%)	175 (67.05%) 76 (29.12%) 10 (3.83%)	0.859	0.598	1.235	0.4113
	G A	475 (83.92%) 91 (16.08%)	426 (81.61%) 96 (18.39%)	0.850	0.620	1.165	0.3123

**Table 4:** Comparison of haplotypes in CHD patients and controls.

Haplotype (R92H/A379V)	Patients (*n* = 566, %)	Controls (*n* = 522, %)	*χ* ^2^	*P* value	OR	95% CI
A-A	3.38 (0.006)	2.10 (0.004)	-	-	-	-
A-G	96.62 (0.171)	65.90 (0.126)	4.285	0.038502	1.428	1.018-2.004
G-A	87.62 (0.155)	93.90 (0.180)	1.198	0.273832	0.837	0.608-1.152
G-G	378.38 (0.669)	360.10 (0.690)	0.504	0.477783	0.911	0.705-1.178

**Table 5:** Logistic regression analysis of Lp-PLA2 gene polymorphisms in CHD.

	Variant	*P* value	OR	95% CI
R92H	AA vs. GG+GA	0.048	4.567	1.015-20.545
V279F	AA vs. AC+CC	0.772	0.690	0.056-8.532
A379V	AA vs. GG+AG	0.805	0.831	0.190-3.626

## Data Availability

The data that support the findings of this study are available from the corresponding author upon reasonable request.

## Abbreviations

CHD: Coronary heart disease
PCR-RFLP: Polymerase chain reaction-restriction fragment length polymorphism.
